# Ductal carcinoma in situ in core needle biopsies and its association with extensive in situ component in the surgical specimen

**DOI:** 10.1186/1755-7682-5-19

**Published:** 2012-06-20

**Authors:** Guerino Barbalaco Neto, Claudia Rossetti, Fernando LA Fonseca, Vitor E Valenti, Luiz Carlos de Abreu

**Affiliations:** 1 Departamento de Mastologia, Faculdade de Medicina do ABC, Avenida Príncipe de Gales, 821, Santo André, SP, 09060-650, Brazil; 2 Departamento de Anatomia Patológica, Faculdade de Medicina do ABC, Avenida Príncipe de Gales, 821, Santo André, SP, 09060-650, Brazil; 3 Departamento de Hematologia e Oncologia, Faculdade de Medicina do ABC, Avenida Príncipe de Gales, 821, Santo André, SP, 09060-650, Brazil; 4 Laboratório de Escrita Científica, Departamento de Morfologia e Fisiologia, Faculdade de Medicina do ABC, Avenida Príncipe de Gales, 821, Santo André, SP, 09060-650, Brazil; 5 Departamento de Fonoaudiologia, Faculdade de Filosofia e Ciências, Universidade Estadual Paulista, Avenida Hygino Muzzi Filho, 747, Marília, SP, 17525-900, Brazil; 6 Departamento de Morfologia e Fisiologia, Faculdade de Medicina do ABC, Av. Príncipe de Gales, 821, Santo André, SP, 09060-650, Brazil

## Abstract

**Background:**

We evaluated the presence of ductal carcinoma in situ (DCIS) in core needle biopsies (CNB) from invasive ductal lesions.

**Methods:**

Retrospective study, which analyzed 90 cases of invasive ductal carcinoma lesions. The percentage of DCIS was quantified in each specimens obtained from CNB, which were compared to the surgical specimens. CNB and surgical specimens were evaluated by the same pathologist, and the percentage of DCIS in CNB was evaluated (percentage) and divided into categories. We considered the following parameters regarding the amount of DCIS: 1 = 0; 2 = 1 for 5%; 3 = 6 for 24%; 4 = 25 for 50%; 5 = 51 for 75% and 6 = 76 for 99%. The number of fragments and the histological pattern of DCIS was found.

**Results:**

We found the following results regarding the distribution of the percentage of DCIS in the CNB: 1 = 63.3%; 2 = 12.2%; 3 = 12.2%; 4 = 5.6%; 5 = 1.1% and 6 = 5.6%. The logistic regression analysis showed that CNB percentages above 45% reflected the presence of DCIS in the surgical specimen in 100% of the cases (p < 0.001), with a specificity of 100%, accuracy of 83.3% and false positive rate of 0% (p <0.001).

**Conclusion:**

There is direct relationship between extensive intraductal component in the surgical specimen when the core biopsy shows 45% or more of the DCI or microinvasive in the material examined.

## Background

Currently, the core needle biopsy (CNB) is a widely used technique which provides better results when compared to needle biopsy [1-5]. Based on a positive diagnosis, this procedure represents a high sensitivity (85% to 98%) and specificity close to 100% with final accuracy of 86% to 97% [6-9]. Moreover, when compared to thinner needle punction, it may distinguish benign and malignant tumors and in situ or invasive forms, mainly due to its ability to obtain malignant histological diagnosis. This method is able to provide important information as prognostic and predictive breast cancer such as histological type, nuclear grade and presence of lymphatic vascular embolization as well as information regarding the biological behavior of tumors where important prognostic factors and treatment are essential, such as the status of estrogen receptors, progesterone receptors, c-erbB-2 markers and also to new therapeutic targets (EGFR, Ki67, etc..), which may be analyzed with the diagnosis, hence, providing a better planning with respect to the therapy to be used [6,10-12].

An important issue in the use of CNB is the possibility of identifying ductal carcinoma in situ (DCIS) associated with invasive lesions. Because the presence of extensive intraductal component (EIC) in breast carcinomas have been associated with increased risk of recurrence in patients treated with conservative surgery in combination with radiation therapy, which would assist in surgical planning [13-19].

Extensive ductal carcinoma in situ (EDCIS) was defined by Schnitt et al [20] when 25% or more of DCIS is present along the invasive lesion. According to Jimenez et al [19], EDCIS in invasive breast carcinomas is associated with increased risk of recurrence in patients treated with conservative surgery and radiotherapy. According to some authors, the status of margins in these cases may represent the greatest predictor of importance for local recurrence. Boyages et al [9] reported a rate of recurrence in the breast of 26% for tumors exhibiting EIC in five years compared to 7% recurrence in negative EIC carcinomas.

In view of the above consideration, this investigation was undertaken to evaluate the presence of DCIS in CNB from invasive ductal lesions and to analysis this relationship.

## Methods

Retrospective study in which 90 cases of invasive ductal carcinoma were investigated. The study was performed in the Breast Cancer Unit of the Department of Clinical Pathology, Faculdade de Medicina do ABC. The specimens corresponded to NOS (not otherwise specified) invasive ductal carcinomas, which corresponds to approximately 75 to 80% of breast carcinomas [21]. Our procedures were approved by the Ethical Committee in Research of the Faculdade de Medicina do ABC (number 109/08).

### Exclusion criteria

We excluded all pure ductal carcinoma in situ or lobular carcinoma in situ (NL3) and invasive lobular neoplasias which exhibited different clinical and radiological features. We also excluded medullary carcinomas which presented in situ component associated [22].

### Inclusion criteria

We selected only ductal carcinomas known to present frank invasion or microinvasion.

### Data collection

In order to collect the specimens we used biopsy gun and 14G needle. We removed between two and six fragments from each specimen, the specimens were processed in a period not exceeding 24 hours. The slides were prepared according to standardization of the Department of Pathology of our Institution, histological sections were performed with a microtome with calibrated thickness of 5 microns, stained with hematoxylin-eosin (HE) and subjected to histological examination with optical microscope Olympus CBA.

The patients were staged according to standards of the Manual of Standards of histopathology reports of the Brazilian Society of Pathology, 2006 [23].

We evaluated the percentage of in situ component observed in each of the specimens from CNB to invasive component, histological pattern (HP) and number of fragments (NF). When the in situ component presented a percentage equal or higher than 25% of the sample it was designated as extensive (EIC), whether in the products of CNB or surgical specimens [24]. In relation to the specimens obtained from CNB, we subdivided into categories, according to the percentage of DCIS: category 1=0; 2=1 for 5%; 3=6 for 24%; 4=25 for 50%; 5=51 for 75% and 6= 76 for 99%. It was also related to patients’ age and to the clinical stage of them at diagnosis.

The data were compared with the surgical specimens for each patient to verify whether the findings in the products of CNB are reflected in their surgical specimens and to evaluate the relationship between the presence and absence of in situ component in CNB when compared to their surgical specimen. Furthermore, we also evaluated its relationship with the presence of EIC and submitted to statistical analysis (univariate and multivariate analysis/logistic regression and ROC curves). All specimens went through the same evaluation criteria by the same pathologist to relate the findings of individual CNB with the specimen from each patient.

### Statistical analysis

Tables were constructed to characterize the findings of the samples. In these tables, categorical variables were presented as absolute frequencies (n) and relative frequencies (%). For analysis of dichotomous or qualitative variables associated with interest group, we used the chi-square or Fisher test. Differences were considered significant when the probability of a Type I error was less than 5% (*p* < 0.05). Variables with p <0.1 in bivariate analysis were included in multivariate analysis by using logistic regression (forward stepwise). We built a model for the evaluation of the findings in CNB and the surgical specimens and correlated it through development of the ROC curve elaboration. In both groups we studied variables such as age, stage, number of fragments and nominal variables: histological type of DCIS when present and the percentage of DCIS in the samples according to the standards range, which were identified by numbers. Statistical calculations were performed with the *Statistical Package for Social Sciences (SPSS)* version 18.0 (2009).

## Results

The distribution of patients according to age was based on suggested intervals according to the Van Nuys Prognostic Index [24], which is used to assess the risk of recurrence of patients according to age at diagnosis of DCIS, with the following results: among 90 cases two (2.2%) were younger than 40 years old, 57 (63.3%) aged between 40 and 60 years old and 31 (34.5%) aged over 60 years old. The mean age of the patients was 57.7 + 15 years old.

Among the 90 patients evaluated, 33 (36.7%) were in situ component. This component ranged from 0 to 90% (average 10.3 + 22%).

Table 1 shows the distribution of patients according to age (category) of the in situ component, the number of fragments and stage.

**Table 1:** Frequency distribution of 90 patients, according to the percentage range of the in situ component, the number of fragments and stage

**Variable**	**Category**	**Group**				**p**
		**Negative (n=67)**		**Positive (n=23)**		
		**N**	**%**	**n**	**%**	
3	< 40 years	2	3	0	0	
Age 2	40 – 60	42	62.7	15	65.2	1^(2)^
Range 1	> 60 years	23	34.3	8	34.8	
		7	10.5			
		9	13.4			
		41	61.2	0	0	
		6	9	4		0.013^(2)^
	T4	2	3	9		
	T3	2	3	3	17.4	
	T2	0	0		39.1	
	T1c				13	
	T1b			1	4.4	
	T1a			4	17.4	
Stage	T1 mic			2	8.7	

We observed in Table 2 that the groups differ with respect to the in situ component in CNB, range and stage. The EIC positive group (23 cases) showed higher percentage of cases with DCIS in CNB compared to the negative group (63 cases). The EIC positive group presented higher percentage of positive cases with the highest percentage of in situ components compared to the negative group. The EIC negative group presented higher percentage of negative cases in T2 and T4, while the EIC positive group presented higher percentage regarding stage, especially in pT1c. We noted that 23 samples of CNB (25.6%) presented EIC in the specimen (Table 2).

**Table 2:** Comparison of positive and negative groups regarding the extensive in situ component in the specimen

**Variable**	**Category**	**Group**				**p**
		**Negative (n=67)**		P**ositive (n=23)**		
		**N**	**%**	**n**	**%**	
	1 - 2	4	6,0	3	13	
	3 - 4	49	73.1	16	69.6	0.539^(1)^
	5 - 6	14	20.9	4	17.4	
	0	51	76,1	6	26.1	
	1 – 5	08	11.9	3	13	
Range (%)	6 – 24	7	10.4	4	17.4	
	26 - 50	1	1.5	4	17.4	< 0,001^(2)^
	51 - 75	0	0	1	4.4	
	76 - 99	0	0	5	21.7	

(1) descriptive level of probability of the Chi-square test.

(2) descriptive level of probability of the Fisher’s exact test.

The groups differed in relation to histological types (Table 3). The positive group presented higher percentage of positive cases in all histological types compared to the negative group. In relation to the multivariate study we used variables significantly associated with the group: in situ CNB, CNB%, stage and four histological types.

**Table 3:** Correlation of overlap between the different histologic patterns identified in the CIE BAG and> 45% in the piece

***Histologic type***	***Comedo***	***Solid***	***Cribriform***	***Micropapilar***	***Papilar***	***Extension to lobe***	***Clinging***
***Comedo***	0	4	4	2	0	2	0
***Solid***	4	0	4	1	0	1	1
***Cribriform***	4	5	0	2	0	1	1
***Micropapilar***	2	1	2	0	0	0	0
***Papilar***	0	0	0	0	0	0	0
***Extension to Lobe***	2	1	1	2	0	0	0
***Cliging***	0	1	1	0	0	0	0

In Table 4 we observed the values of the estimated probability, odds ratios and indices of efficiency of logistic regression model.

**Table 4:** Values of the estimated probability, odds ratios and indices of efficiency of logistic regression model

***CNB (%) of DCIS***	***Probability***	***Odds***	***IC to 95%***							
		***ratio***	***LL***	***UL***	***S***	***E***	***FP***	***FN***	***Accuracy***	***p***
0	0.117	-	-	-	-	-	-	-	-	-
5	0.180	9.03	3.05	26.78	73.9	76.1	48.5	10.5	75.6	< 0.001
10	0.265	11.47	3.77	35.04	60.9	88.1	36.4	13.2	81.1	< 0.001
15	0.374	42.25	8.27	215.77	56.5	97	13.3	13.3	86.7	< 0.001
20	0.496	42.25	8.27	215.77	56.5	97	13.3	13.3	86.7	< 0.001
25	0.619	60.50	7.14	512.94	47.8	98.5	8.3	15.4	85.6	< 0.001
30	0.728	50.77	5.97	431.48	43.5	98.5	9.1	16.5	84.4	< 0.001
35	0.815	42.43	4.97	362.41	39.1	98.5	10	17.5	83.3	< 0.001
40	0.879	42.43	4.97	362.41	39.1	98.5	10	17.5	83.3	< 0.001
45	0.923	-	-	-	34.8	100	0	18.3	83.3	< 0.001
50	0.952	-	-	-	34.8	100	0	18.3	83.3	< 0.001
55	0.970	-	-	-	34.8	100	0	18.3	83.3	< 0.001
60	0.982	-	-	-	34.8	100	0	18.3	83.3	< 0.001
65	0.989	-	-	-	34.8	100	0	18.3	83.3	< 0.001
70	0.993	-	-	-	34.8	100	0	18.3	83.3	< 0.001
75	0.996	-	-	-	34.8	100	0	18.3	83.3	< 0.001
80	0.998	-	-	-	34.8	100	0	18.3	83.3	< 0.001
85	0.998	-	-	-	34.8	100	0	18.3	83.3	< 0.001
90	0.999	-	-	-	34.8	100	0	18.3	83.3	< 0.001

Sensitivity (S), specificity (E), False Positive (FP), False Negatives (FN), Lower Limit (LL) Upper Limit (UL).

**Figure 1:**
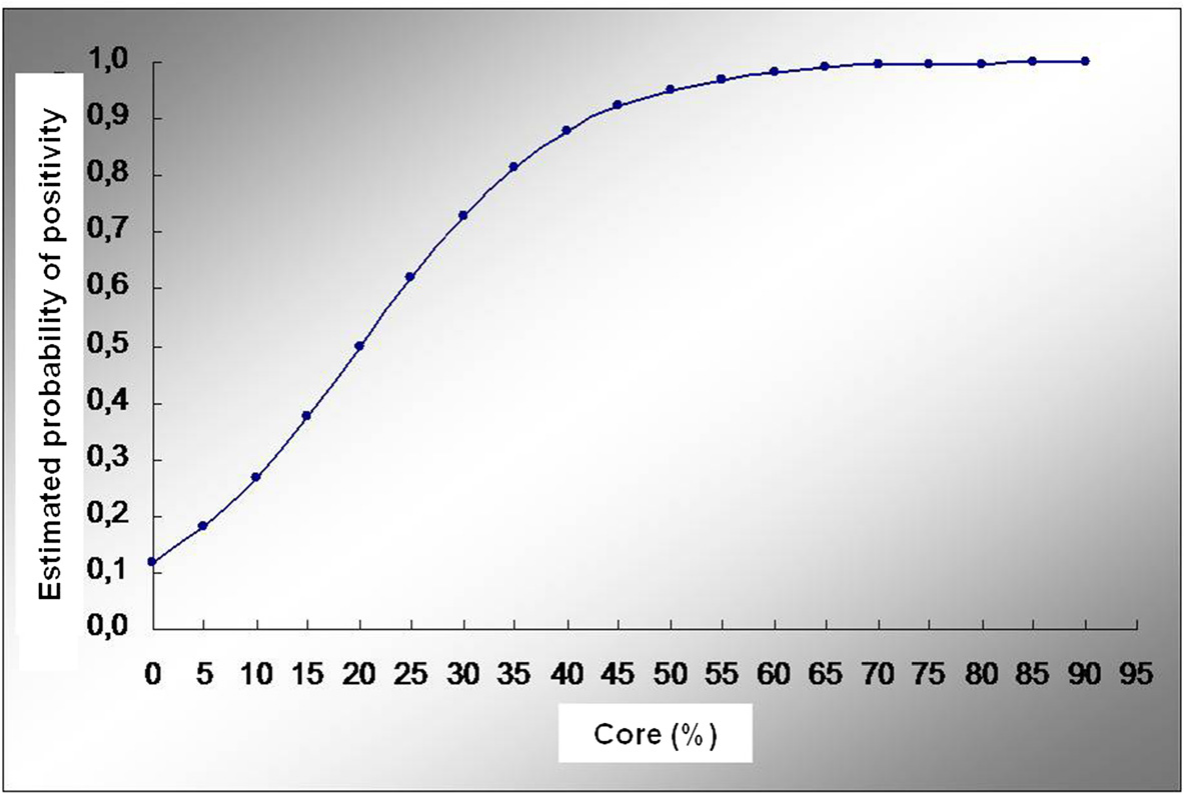
**Logistic curve.** Log (p/1-p) = -2.019 + 0.101% *Core%. p = probability of the patient to be positive.

**Figure 2:**
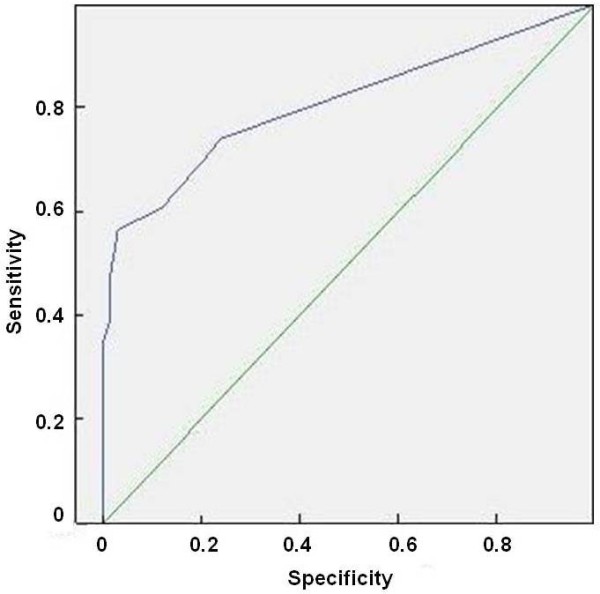
ROC curve for the% of DCIS found in the specimen.

Figure 1 and Figure 2 present data regarding statistical analysis. We reported logistic curve and ROC curve for the% of DCIS found in the specimen.

## Discussion

In this study we evaluated the presence of DCIS in CNB from invasive ductal lesions. Our results demonstrate that there is relationship between DCIS in the surgical specimen and 45% or more of the in situ component in CNB.

Previous studies indicated that EIC in breast carcinomas was associated with an increased risk of recurrence in patients treated with conservative surgery and radiotherapy [13-17]. EIC was identified as a risk factor for local recurrence following lumpectomy since 1984 [20]. Based on our report, the risk of local recurrence for high-grade invasive tumors was 4% to 39% when EIC was present.

Although the 12-gauge CNB presents a tissue volume about five times larger than the 14-gauge (this form would present higher chances to represent the tumor profile besides its relatively cheap price), some information regarding the tumor size is sometimes compromised. It leads some authors to prefer the 14-gauge needle biopsy, which is standardized in our department. Thus, we used 14-gauge needles, as recommended by the literature. On the other hand, we found no correlation with the number of fragments provided for diagnosis, because approximately 65 cases (72.2%) were composed of 3–4 fragments, compared with 15 cases (20% of the sample), which were sent 5–6 fragments per specimen for analysis, opposite to the results found by Parker et al [2], Liberman et al [21], Abreu-e-Lima et al [25] and Abreu-e-Lima et al [26].

CNB analysis in which we did not identify DCIS was associated with a higher likelihood of clear margins (15% versus 24%). Some studies tried to demonstrate the relationship between DCIS in CNB and the presence of the in situ component in extensive surgical specimens in order to prevent the deterioration of margins in conservative surgery. Mai et al [18] evaluated 78 CNB and compared it to surgical specimens in an attempt to predict the presence of EIC in the specimens. They noted that there was good correlation between DCIS and CNB, the CNB showed the presence of at least three foci of low-grade DCIS or at least two foci of high grade DCIS and revealed the presence of compromised margins on products of lumpectomy.

Our investigation was not based on the number of foci identified regarding the presence of DCIS, it was based on an analysis that seemed to be of easy applicability, which was composed by the assessment of the percentage of DCIS found in CNB and its relationship with EIC in the surgical specimen. EIC has been implicated as an independent predictor of recurrence in breast carcinomas treated with conservative surgery and radiotherapy. In 1990, Boyages et al [13], through a study with a follow up of five years showed recurrence in breast tissue in 26% of cases with EIC versus 7% compared to those who did not present this component. Moreover, the presence of EIC in lumpectomy products correlated with extensive residual disease in unresected tissues. Dzierzanowski et al [27] evaluated 95 cases of patients diagnosed with invasive carcinoma and its correlation with extensive in situ component in conservative surgeries. Their selection, however, included cases of pure DCIS and invasive in CNB, where only 34 cases (79%) exhibited CNB associated with the invasive component. In order to avoid a bias towards overestimating the presence of DCIS in CNB compared to the specimen, we selected cases with frank invasion. In this context, Dzierzanowski et al [27] concluded that the presence of pure DCIS in CNB specimens when correlated with the invasive lesion showed a greater risk for positive margins. Nevertheless, the percentage of DCIS in CNB expected for such a conclusion was not well clarified.

In order to evaluate the correlation between DCIS in CNB and the presence of EIC, we assessed whether there would be a percentage that reflects a relationship between the presence of in situ component in the CNB with this finding in surgical specimens. We observed through the logistic regression analysis that the percentages of DCIS in CNB above 45% reflected the presence of the in situ component in the surgical specimen in 100% of cases with a specificity of 100%, accuracy of 83.3% and false positive rate of 0% (p <0.001). Percentage values of DCIS in CNB between 15 and 20% showed a sensitivity of 56.5%, specificity of 97%, accuracy of 86.7% and 13.3% false positive (p <0.001).

With respect to the histological type presented by the DCIS in CNB, it was not relevant based on the presence of EIC in the surgical specimen. It suggests that the histological type of DCIS presented in the CNB, even when dealing with the standard micropapillary, which exhibits high correlation with multifocality in breast lesions, is not associated with the presence of EIC in the surgical specimen.

EIC has been implicated as an independent predictor of recurrence in breast carcinomas treated with conservative surgery and radiotherapy. In patients with invasive carcinoma, the risk of metastatic disease is already present at diagnosis and many failures can occur without evidence of local recurrence, but in DCIS the risk of metastases at diagnosis is negligible. Therefore, an invasive local recurrence brings the possibility of death from breast cancer, especially when conservative surgery for treatment is used [28,29].

In our study, stage related to higher percentage of positivity for EIC corresponded to PT1C. This fact leads us to suggest that invasive tumors that measured between 1 and 2 cm exhibit a higher probability to be associated with intraductal component, unlike lesions in more advanced stages, i.e., stage pT4, where the component has already acquired earlier in situ invasive profile and is less often found in these lesions.

In a study of Holland et al [17], 71% of cases with positive EIC in the diagnosis of CNB showed residual DCIS in subsequent mastectomy product compared to 28% of cases with negative EIC. Similarly, Schnitt et al [20] reported that 88% of DCIS cases showed residual positive EIC re-excision in cases when compared with negative EIC. It suggests that the distribution of EIC associated with tumors has been underestimated in their contribution to surgical planning. It has been demonstrated in the conservative treatment for breast cancer with EIC, where there is high recurrence rate which is likely due to the presence of residual lesion of DCIS [13]. Holland et al [17] showed that invasive breast tumors with positive CIE have a greater likelihood to present residual DCIS compared to negative EIC tumors. EIC was found in 30% - 40% of all invasive breast tumors. Due to the fact that margin status corresponds to the strongest predictor of local recurrence, such information seems to be an important predictive value when adopting techniques of conservative treatment, and the anticipation of this information whenever possible is a important tool for appropriate surgical planning.

This study was undertaken to characterize in a less invasive diagnostic technique (CNB) the predictive ability in relation to the presence of EIC in the surgical specimens, warning prior to the possible involvement of surgical margins in conservative surgery, showing, if possible, the best parameter to be adopted in these specimens for the characterization of EIC. Therefore, proposing a cutoff for the truly significant presence that helps the surgical approach with lower risk of recurrence. Through this study we suggest the percentage of DCIS in the CNB to be mentioned in reports of pathology, thereby allowing better surgical planning. Furthermore, tumors are a clinical condition worth to be investigated [30-33].

Our study present a point that should be addressed, one may argue that the diagnostic enthusiasm for the high specificity may be moderated by the fact that CNBs was obtained from excised specimens and not from patients. We evaluated only invasive carcinoma and the presence of intraductal invasion in these cases is very frequent. Nonetheless, we may consider excided specimen as a consistent method, because a previous report indicated the importance of complete excision in the prevention of local recurrence of ductal carcinoma in situ [17].

## Conclusion

There is a direct relationship between the percentage of DCIS present in DCI or microinvasive obtained by CNB and EIC in the surgical specimen.

## Abbreviation

CNB, Core needle biopsy; DCIS, Ductal carcinoma in situ; EIC, Extensive intraductal component; EDCIS, Extensive ductal carcinoma in situ; HE, Hematoxylin-eosin; HP, Histological pattern; NF, Number of fragments; S, Sensitivity (S); E, Specificity; FP, False positive; FN, False negatives.

## Competing interests

We declare no conflict of interest.

## Authors’ contribution

All authors participated in the acquisition of data and revision of the manuscript. GBN, CR, FLAF, VEV and LCA conceived of the study, determined the design, performed the statistical analysis, interpreted the data and drafted the manuscript. All authors read and gave final approval for the version submitted for publication.
